# Skin Cancer Recognition and Education Among Community Health Workers in Southern New Mexico

**DOI:** 10.3390/ijerph23080994

**Published:** 2026-07-29

**Authors:** Simran D. Bhakta, Tyler S. Peters, Alessandra Merenna, Debra E. Bramblett

**Affiliations:** Burrell College of Health Sciences, 3501 Arrowhead Dr, Las Cruces, NM 88001, USA; alessandra.merenna@burrell.edu (A.M.); dbramblett@burrell.edu (D.E.B.)

**Keywords:** community health workers, skin cancer, education, New Mexico, promotoras, melanoma, prevention, survey

## Abstract

**Highlights:**

**Public health relevance—How does this work relate to a public health issue?**

- Community health workers play an important role in improving access to health education and promoting early detection of skin cancer in underserved communities.
- This study evaluated the effectiveness of a brief educational intervention to improve skin cancer recognition knowledge among community health workers in southern New Mexico.

**Public health significance—Why is this work of significance to public health?**

- A short educational intervention significantly improved community health workers’ confidence and comfort in recognizing skin lesions suspicious for melanoma.
- Enhancing community health worker education may strengthen community-based efforts to promote earlier recognition of skin cancer and timely referral for evaluation.

**Public health implications—What are the key implications or messages for practitioners, policy makers and/or researchers in public health?**

- Incorporating standardized skin cancer education into community health worker training programs may improve public awareness and support earlier detection of skin cancer.
- Future studies should evaluate whether improvements in knowledge translate into increased community education, appropriate referrals, and earlier skin cancer diagnosis.

**Abstract:**

NM averages 450 new cases of melanoma annually. Promotoras are community health workers in NM that provide health education and resources to the community. The project aims to determine if promotora education can improve the recognition of suspicious lesions, encouraging community members to seek care. Current literature focuses on the self-reported confidence of CHWs in recognizing skin cancer. A total of 54 promotoras were sampled through a Qualtrics quiz asking whether skin lesion photos are suspicious. Participants were also asked to rate their confidence in recognizing a suspicious skin lesion and comfort in addressing it from 1 to 5. Participants then read an educational flyer about the “ABCDEs” of melanoma before repeating the quiz/ratings. Wilcoxon signed-rank testing revealed significant increases in quiz scores (*W* = 220.5, *p* = 0.045), confidence (*W* = 44.0, *p* < 0.001), and comfort (*W* = 8.5, *p* < 0.001). Promotoras could be an additional line of defense against melanoma, helping save lives. Healthcare personnel with limited skin lesion knowledge could be an ideal future population for education.

## 1. Introduction

The purpose of this project is to investigate the role that community health workers can play in skin health awareness with a particular emphasis on melanoma. Melanoma is a skin cancer of pigment-producing cells called melanocytes and has been expertly reviewed elsewhere [1,2]. Consistent sun exposure, a major risk factor for the development of melanoma, is not avoidable for a sizable portion of the New Mexico population. There are 20,976 farms in New Mexico operated by more than 37,000 individuals [3], all of whom are exposed to potentially harmful UV radiation through their work. This exposure dramatically elevates the likelihood of developing melanoma.

New Mexico had an average of 450 new cases of melanoma each year from 2017 to 2021 [4] and 15% of those cases were detected late [5]. Early detection of melanoma significantly improves cure rates, with one study showing an over 70% case fatality rate for melanomas detected late, defined as thicker than 3.6 mm, compared to a 96% cure rate for melanomas thinner than 0.76 mm [6]. Though melanoma can be alarming, being aware of the definitive signs of this skin cancer can encourage those with suspicious lesions to seek out medical consultation from a dermatologist.

In Southern New Mexico, community health workers (CHWs) known as ‘promotoras’, or promotores de Salude (Health Promoters), provide basic health education, earning a certificate after an 80-h training program of healthcare navigation and disease prevention [7]. Promotoras are especially valuable in areas of low-income or predominantly Spanish-speaking individuals, serving as a liaison between a community member and a physician. This project aims to familiarize promotoras with the ABCDEs of melanoma detection and utilize their influence to recognize suspicious lesions and advise potentially affected community members to visit a healthcare professional.

Current literature about the role of promotoras in skin cancer recognition focuses on the self-reported confidence of CHWs in educating individuals about signs of melanoma, which was found to be 82.4% of the 19 promotoras involved in one study [8]. However, no studies actually test this newfound confidence, which our study aims to accomplish.

## 2. Materials and Methods

Sampling was conducted in Doña Ana County, New Mexico through a combined quiz and survey created and administered electronically via the Qualtrics platform (Provo, UT, USA). Consent forms and the Qualtrics survey/quiz were distributed to all 160 promotoras in Doña Ana County via email. Each promotora was given the opportunity to participate in the project by completing the survey, viewing educational material, and taking a pre-/post-quiz on a completely voluntary basis without incentive. The only inclusion criteria for subjects was completion of promotora training and serving in Doña Ana County. A total of 54 promotoras completed the quiz, giving a response rate of 33.75%.

The Qualtrics survey/quiz was offered in both English and Spanish, giving participants a choice of language preference. A consent form, also both in English and Spanish, was presented at the beginning of the quiz and required signature for completion and inclusion in the data. The Qualtrics Survey was used to collect demographic information, and all responses were required.

To gauge the ability of promotoras to effectively recognize suspicious lesions, this study involved a 10-question pre-/post-quiz given before and after viewing an educational flyer about melanoma warning signs. Each quiz question asks whether or not a photo of a skin lesion is suspicious. The gross photos used for the quiz were obtained from DermNet Images (dermnetnz.org), seen in Appendix A, and used in accordance with the licensing requirement of Creative Commons Attribution NonCommericial-NoDerivs 3.0. A board-certified dermatologist, mentioned in the acknowledgements, helped select, review, and approve the use of these 10 images. The 10 images vary in composition and difficulty, addressing some or multiple aspects of the educational flyer. Participants were also asked to rate their confidence in recognizing a suspicious skin lesion on a scale from 1 to 5 with 5 being the most confident, as well their comfort in speaking to a community member about the suspicious skin lesion, also on a scale from 1 to 5 with 5 being the most comfortable.

**Figure 1:**
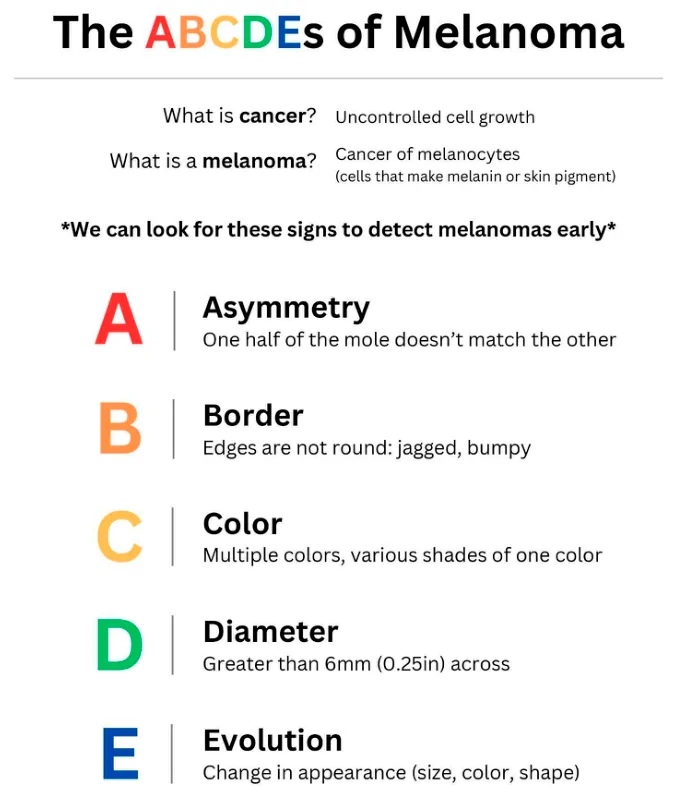
ABCDEs of melanoma. Educational flyer presented to the promotoras in between the pre- and post-quiz. This figure was created in collaboration with a board-certified dermatologist and reflects five main clues for identifying lesions suspicious of melanoma.

Following completion of the pre-quiz, participants were instructed to read the educational flyer (Figure 1) about the “ABCDEs” of melanoma, highlighting the specific warning signs of asymmetry, border, color, diameter, and evolution. Specifically, participants were instructed to look for asymmetry by noting differences in halves of the lesion, borders with non-round edges, multiple colors or shades of one color, and diameters greater than 6 mm (0.25 inch). Evolution was not tested in this quiz as only 1 image was presented per question and changes in appearance over time could not be measured.

Participants were then asked to take a follow-up quiz with the same questions as the first quiz in a different order, including the 10 suspicious skin lesions and questions to reassess the respondent’s comfort with communicating with patients about skin lesions and confidence in identifying suspicious lesions. The Burrell College of Health Sciences Institutional Review Board approved this study.

Pre- and post-educational flyer quiz scores, confidence levels, and comfort levels were compared using the Wilcoxon signed-rank test due to the paired nature of the data. All statistical analyses were performed using jamovi (Version 2.6), an open- source statistical software [9]. Statistical significance was defined as *p* < 0.05. Descriptive statistics are reported as median and the interquartile range (IQR = 25th–75th percentiles). Quartiles were calculated using the R-7 method within jamovi. Lastly, an effect size (*r* = Z/√N) was calculated per variable. Following the educational flyer, participants demonstrated statically significant improvements in knowledge, confidence, and comfort in addressing lesions suspicious for melanoma.

## 3. Results

After reviewing the promotoras’ performance, the average score on the pre-test was found to be 5.91 questions correct out of 10. Once the promotoras reviewed the educational flyer, the average post-quiz score was 6.35 questions correct out of 10, showing an average improvement of 0.44 correct questions.

Table 1 defines the hypotheses and terms for this study. Wilcoxon signed-rank testing revealed significant increases in quiz scores between the pre- and post-quiz (*W* = 220.5, *p* = 0.045). With a sample size of 54 and a significance (α) of 0.05, the *p*-value was calculated to be 0.045 (Table 2). Since *p* < α, the null-hypothesis (H_0_) is rejected and we conclude the difference in pre- and post-quiz scores is statistically significant, with a moderate effect size (*r* = 0.373). Promotoras were able to recognize melanoma better after the educational flyer was presented.

Similarly, a second and a third Wilcoxon signed-rank tests were used to examine the difference in average confidence (*W* = 44.0, *p* < 0.001, *r* = 0.749) and comfort ratings (*W* = 8.5, *p* < 0.001, *r* = 0.911), with both demonstrating large effect sizes as seen in Table 2. The difference in pre- and post-confidence ratings was determined to be statistically significant, an important element of community interaction supporting the likelihood of these promotoras actually speaking to an individual if said findings were present.

**Figure 2:**
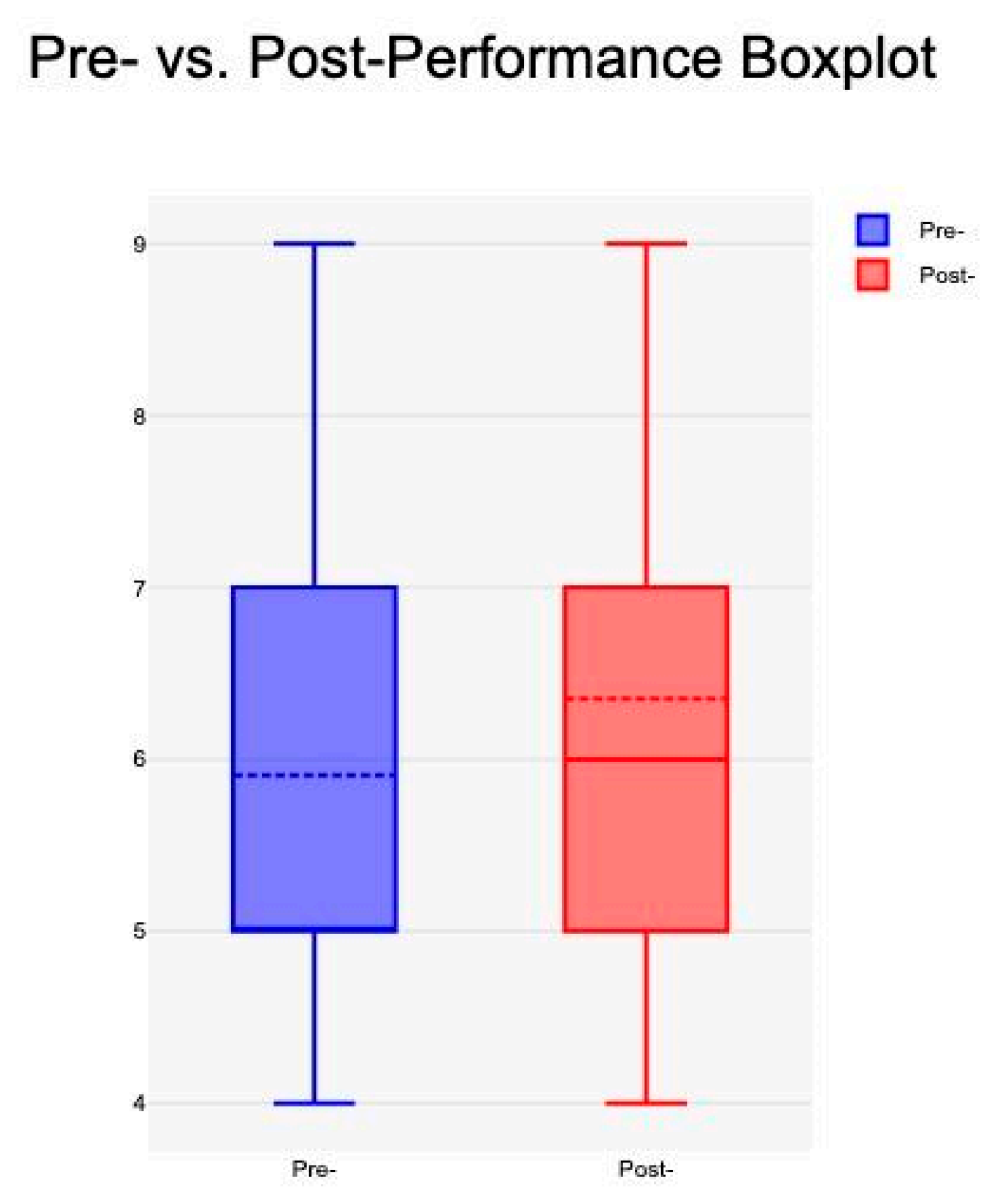
Pre- and post-absolute difference boxplot. Box plot of absolute differences between pre- and post-quiz performance. The solid line represents the median for each data set, and the dotted line represents the mean [10].

**Figure 3:**
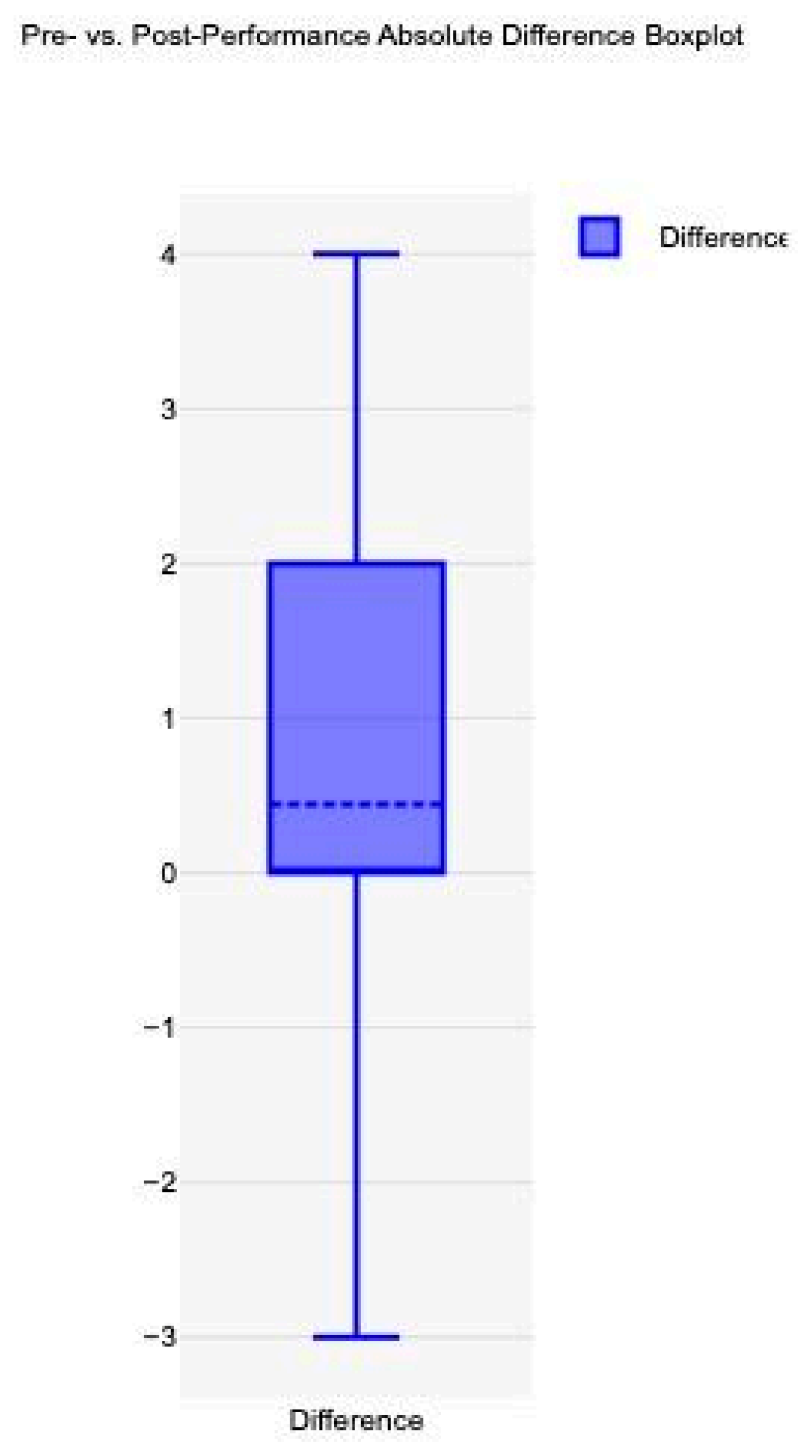
Pre- and Post-absolute difference boxplot. Box plot of absolute differences between pre- and post-quiz performance. The dotted line represents the mean [10].

Lastly, Figure 2 and Figure 3 show the absolute difference in performance pre- and post-quiz. Figure 2 shows an increase in the median absolute difference (pre-quiz = 5, post-quiz = 6) between the pre- and post-performances, and Figure 3 shows a mean absolute increase of 0.44 questions correct between pre- and post-performance. Reading this simple educational flyer resulted in an overall improvement in recognition of melanoma. The practical relevance of this small increase is that a basic educational tool can increase the likelihood of skin cancer detection and subsequent intervention.

**Figure 4:**
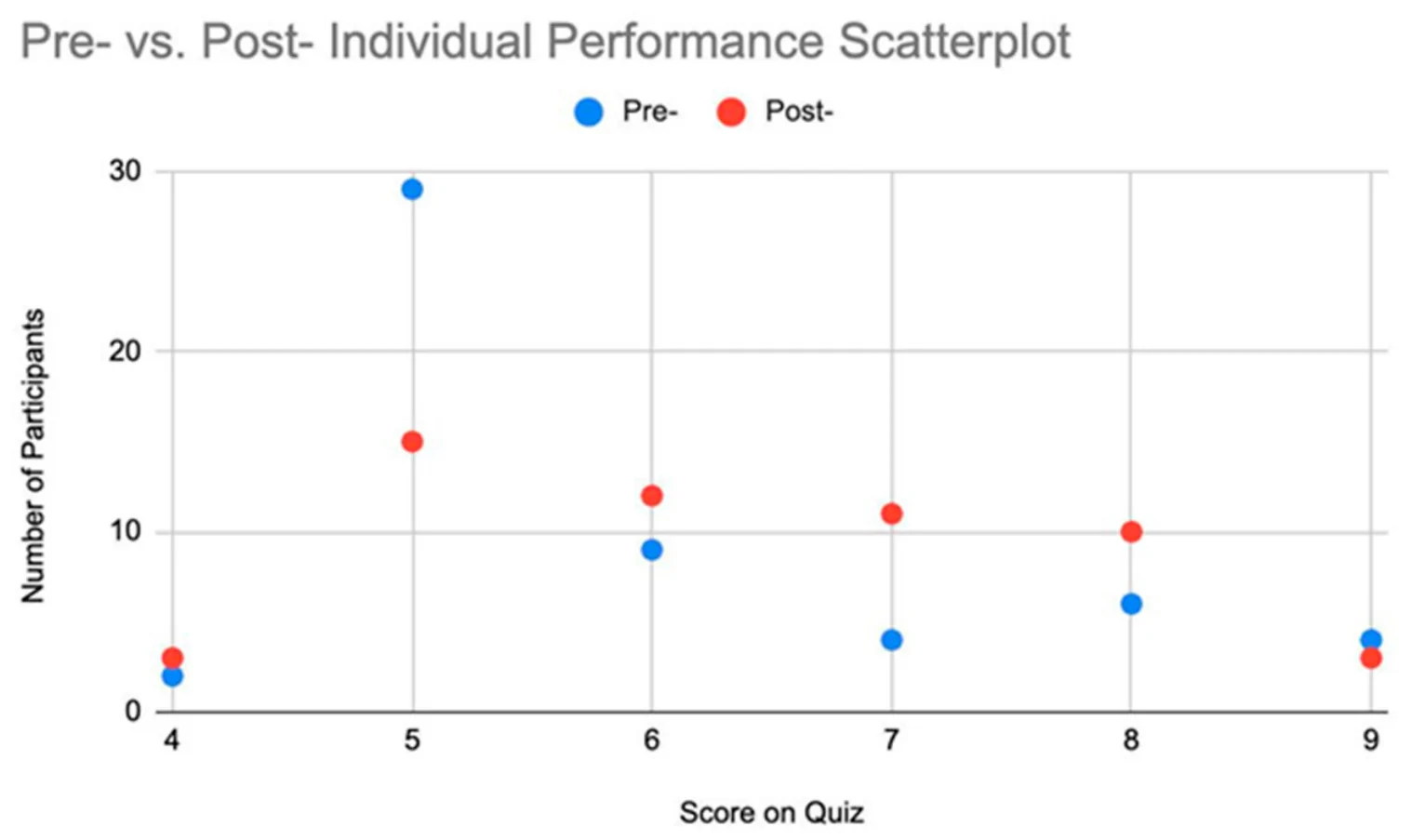
Pre- versus post-individual performance scatterplot of score frequencies. Distribution of pre- and post-intervention quiz scores. Blue markers represent the frequency of each pre-intervention quiz score. Red markers represent the frequency of each post-intervention quiz score.

Furthermore, Figure 4 demonstrates the distribution of quiz scores before and after participants reviewed the educational flyer. Figure 4 illustrates a rightward shift in the distribution of quiz scores. Compared with baseline, fewer participants scored 5/9, while more participants achieved scores of 6–8/9 after reviewing the educational material. This suggests improved knowledge after the educational material and remains consistent with the significant improvement observed in the Wilcoxon signed-rank analysis.

## 4. Discussion

This study demonstrates that participation in a brief educational intervention was associated with modest but statistically significant improvements in CHW knowledge, confidence, and comfort regarding melanoma identification and prevention in the Doña Ana community of southern New Mexico. Utilizing promotoras illustrates a new application of survey-based research with the potential to improve comprehension and delivery of medical information to the general public.

These findings are consistent with previous studies demonstrating that structured melanoma education can improve confidence and melanoma recognition skills. Heshmati et al. found that educational interventions with CHWs helped improve self-efficacy and communication-related outcomes [11]. This supports the importance of structured interventions when training promotoras. Additionally, melanoma-focused education has been proven to improve recognition of suspicious lesions. Robinson et al. found that training individuals on ABCDE criteria improved recognition of melanoma-associated features [12]. This supports the role of the ABCDE-based educational flyer in improving perceived melanoma recognition skills. Together, these findings suggest that a brief and focused melanoma educational flyer may be a practical strategy for improving promotora short-term educational outcomes and perceived preparedness.

Although the increase in quiz performance was statistically significant, the absolute improvement was modest, with participants answering fewer than one additional question correctly on the 10-item assessment after the intervention. Therefore, these findings should be interpreted as evidence of improved knowledge acquisition, and suggest that brief educational materials may represent a feasible first step toward improving melanoma awareness among promotoras. Additional research is needed to determine whether these improvements are retained over time and translate into meaningful changes in real-world melanoma recognition or patient outcomes. Given the substantial burden of melanoma, small improvements in awareness may justify further investigation into the clinical outcomes of this intervention [13]. Moreover, many do not call attention to suspicious lesions they may have for many reasons, including lack of access to care, fear of the healthcare system, or even mistrust of the healthcare professional. This study addresses many of these factors as promotoras are well-trusted members of the community and provide a needed bridge from patient to provider. Promotoras are also accessible healthcare resources who may help promote earlier recognition of suspicious skin lesions and encourage timely medical evaluation when provided with appropriate training. As this was a brief, low-cost educational intervention requiring minimal resources, whether repeated or more comprehensive educational programs produce larger and clinically meaningful improvements warrants further investigation.

Similarly, although statistically significant improvements in confidence and comfort were observed, the magnitude of these changes was relatively small. These findings suggest that the educational intervention positively influenced participants’ perceived preparedness. However, changes in self-reported confidence should not be interpreted as evidence of improved clinical competency or behavior. Longitudinal evaluation will be needed to determine whether these gains are sustained over time and influence patient education, referral patterns, or earlier detection of suspicious skin lesions.

A challenge we faced when implementing this education training program was that the quiz was delivered remotely, which did not enable us to track time spent on the quiz or control the time of day or setting in which the quiz was taken. In the future, this quiz could be delivered in person to better control for these variables, particularly setting a cut-off for the time spent on the quiz and controlling the setting in which all promotoras must take the quiz. This study was limited by the fact that we did not inquire about past educational experiences relating to skin health. Also, this study pertained only to knowledge regarding melanoma and not other skin lesions. One limitation includes the format of an online quiz and the flyer lacking demonstration of skin lesion changes over time. The response rate of the survey was low (33.75%) and may indicate a selection bias. However, we feel the 66.25% refusal rate may reflect reduced access to appropriate electronic devices of promotoras, a lack of free time to complete the survey, a lack of familiarity with the name of our institution on the email, or the manner in which we recruited subjects. However, selection bias remains a limitation, as promotoras who participated may have differed systematically from those who did not participate. For example, some participants may have had greater interest in self-education which may limit the generalizability of our findings to the broader promotora population. That being said, the average response of an online survey ranges from 2% to 30% [14] and the response to our study was notably higher. Thus, we feel confident that this represents a good cross-section of the promotora cohort in Dona Aña County. Another limitation may be that this study measured immediate post-intervention knowledge and self-reported attitudes rather than long-term knowledge retention or actual melanoma recognition in clinical or community settings. A final limitation may be that the impact of this educational intervention was larger on a subset of participants compared to the whole. This may be because we did not document the level of experience of each promotora, any prior training in this area, or personal experience with skin cancer. Subsequent studies with promotoras should include these elements to better understand how prior experience influences educational outcomes. Consequently, the practical significance of the observed improvements remains uncertain.

Further investigation of promotoras’ past experience with advocating for patients with skin care problems could guide our understanding of the significance of these findings. Additional work should involve broader training in skin health issues, including basal cell carcinoma, squamous cell carcinoma, and infectious skin disease. Future work should determine whether educational interventions among these groups improve skin lesion recognition, referral behavior, and ultimately patient outcomes.

## 5. Conclusions

Skin care in New Mexico is critical to public health. With the increased UV index in this region, melanoma detection and treatment can help save lives. Promotoras play a vital role in connecting the public to physicians to detect and treat skin cancer. If promotoras engage in foundational dermatological training to identify suspicious skin lesions, they have the potential of becoming the frontline in the battle against the progression of serious skin conditions such as melanoma.

## Figures and Tables

**Table 1:** Hypotheses and key terms.

Sample Size	54 Promotoras
Pre-	Score obtained/rating marked before reading the educational flyer
Post-	Score obtained/rating marked after reading the educational flyer
H_0_	There is no significant difference between the average pre- and post-quiz scores; there is no significant difference between the average pre- and post-confidence levels; there is no significant difference between the average pre- and post-comfort levels
H_a_	There is a significant difference between the average pre-and post-quiz scores; there is a significant difference between the average pre- and post-confidence levels; there is a significant difference between the average pre- and post-comfort levels

**Table 2:** Wilcoxon-signed-rank tests with descriptive statistics.

Outcome	Quiz Score	Confidence	Comfort
Pre-quiz Median (Q1–Q2) *	5 (5.0–6.5)	3.0 (2.0–4.0)	3.0 (2.0–4.0)
Post-quiz Median (IQR) *	6 (5.0–7.0)	3.0 (2.25–4.0)	3.5 (3.0–4.75)
Wilcoxon *W*	220.5	44.0	8.5
*p* value	0.045	<0.001	<0.001
Effect size (*r* = Z/√N)	0.373	0.749	0.911

* Values in parentheses in the outcome column are presented in parentheses in the quiz score, confidence, and comfort columns accordingly.

## Data Availability

Data can be made available upon request to authors.

## Appendix A

Photos utilized in the pre- and post- quiz. Each photo contained the caption “Is this suspicious?” with the answer choices of Yes or No. Images sourced from DermNet New Zealand under the DermNet Image Licence (CC BY-NC-ND 4.0). Watermarks retained. Image sources: melanonoma (upper left) Reprinted with permission from Ref [15]. Copyright © n.d. DermNet New Zealand Trust; nevus (upper right) Reprinted with permission from Ref [16]. Copyright © 2007 DermNet New Zealand Trust; nevus (lower left) Reprinted with permission from Ref [17]. Copyright © 2007 DermNet New Zealand Trust; superficial spreading melanoma (lower right) Reprinted with permission from Ref [18]. Copyright © DermNet New Zealand Trust.
